# UCapsNet: A Two-Stage Deep Learning Model Using U-Net and Capsule Network for Breast Cancer Segmentation and Classification in Ultrasound Imaging

**DOI:** 10.3390/cancers16223777

**Published:** 2024-11-09

**Authors:** Golla Madhu, Avinash Meher Bonasi, Sandeep Kautish, Abdulaziz S. Almazyad, Ali Wagdy Mohamed, Frank Werner, Mehdi Hosseinzadeh, Mohammad Shokouhifar

**Affiliations:** 1 Department of Information Technology, Vallurupalli Nageswara Rao Vignana Jyothi Institute of Engineering and Technology, Hyderabad 500090, India; madhu_g@vnrvjiet.in (G.M.); avinash.bonasi@gmail.com (A.M.B.); 2 APEX CSE, Chandigarh University, Mohali 140403, India; dr.skautish@gmail.com; 3 Department of Computer Engineering, College of Computer and Information Sciences, King Saud University, Riyadh 11543, Saudi Arabia; mazyad@ksu.edu.sa; 4 Operations Research Department, Faculty of Graduate Studies for Statistical Research, Cairo University, Giza 12613, Egypt; aliwagdy@gmail.com; 5 Applied Science Research Center, Applied Science Private University, Amman 11931, Jordan; 6 Faculty of Mathematics, Otto-von-Guericke University, 39016 Magdeburg, Germany; 7 School of Computer Science, Duy Tan University, Da Nang 550000, Vietnam; 8 Jadara University Research Center, Jadara University, Irbid 21110, Jordan; 9 DTU AI and Data Science Hub (DAIDASH), Duy Tan University, Da Nang 550000, Vietnam; mohammadshokouhifar@duytan.edu.vn

**Keywords:** breast cancer, ultrasound imaging, segmentation, classification, U-Net, capsule network

## Abstract

**Background/Objectives**: Breast cancer remains one of the biggest health challenges for women worldwide, and early detection can be truly lifesaving. Although ultrasound imaging is commonly used to detect tumors, the images are not always of sufficient quality, and, thus, traditional U-Net models often miss the finer details needed for accurate detection. This outcome can result in lower accuracy, making early and precise diagnosis more difficult. **Methods**: This study presents an enhanced U-Net model integrated with a Capsule Network (called UCapsNet) to overcome the limitations of conventional techniques. Our approach improves segmentation by leveraging higher filter counts and skip connections, while the capsule network enhances classification by preserving spatial hierarchies through dynamic routing. The proposed UCapsNet model operates in two stages: first, it segments tumor regions using an enhanced U-Net, followed by a classification of the segmented images with the capsule network. **Results**: We have tested our model against well-known pre-trained models, including VGG-19, DenseNet, MobileNet, ResNet-50, and Xception. By properly addressing the limitations found in previous studies and using a capsule network trained on the Breast Ultrasound Image (BUSI) dataset, our model resulted in top-achieving impressive precision, recall, and accuracy rates of 98.12%, 99.52%, and 99.22%, respectively. **Conclusions:** By combining the U-Net’s powerful segmentation capabilities with the capsule network’s high classification accuracy, UCapsNet boosts diagnostic precision and addresses key weaknesses in existing methods. The findings demonstrate that the proposed model is not only more effective in detecting tumors but also more reliable for practical applications in clinical settings.

## Simple Summary

Current ultrasound imaging methods for detecting breast cancer often face challenges with image quality, making it tough to accurately spot tumors. This paper introduces UCapsNet, a new model that combines two advanced techniques to enhance breast cancer detection in ultrasound images. By improving how tumors are segmented and classified, UCapsNet aims to deliver clearer and more accurate results compared to traditional methods. Our findings indicate that UCapsNet can greatly improve diagnostic precision, enabling earlier detection and better treatment options for patients. This research could significantly impact future studies and lead to improved breast cancer detection practices in the medical community.

## 1. Introduction

Breast cancer is one of the most common cancers worldwide, affecting women across the globe. In 2022, it claimed nearly 670,000 lives. Each year, an astounding 2.3 million women—over a quarter of all female cancer patients globally—are diagnosed with breast cancer [1]. By 2040, the incidence of breast cancer is expected to increase by over 40%, reaching approximately 3 million cases annually, driven by factors such as population growth and aging. Similarly, breast cancer-related deaths are projected to rise by more than 50%, reaching around 1 million annually by 2040 [2]. Breast cancer primarily affects the inner layers of milk glands or lobules and ducts, which are tiny tubes that transport milk [3]. The timely recognition of breast cancer plays a critical role in securing a positive projection and achieving survival rates of high magnitude. Noteworthy is the fact that, in North America, the relative survival rate of 5 years for patients suffering from breast cancer exceeds over 80%, thanks to the critical impact of quick affliction detection [4]. The global imperative to enhance breast cancer survival rates depends significantly on early detection. Survival rates are increased, and treatment costs are lowered when ultrasound imagery is used in early breast cancer detection [5,6,7,8].

Segmentation techniques for tumor detection involves extracting features, detection, treatment, and classification stages [9]. For differentiating malignant from benign breast cancer, region of interest (ROI) extraction is used as the initial phase. To enhance the accuracy of this crucial step and minimize false positives, a novel method based on local pixel data and neural networks is introduced [10]. After segmentation, the next step is breast cancer classification, which typically utilizes structural and texture features of the ultrasound images. Usually, these features are used to determine whether the tumor is malignant or benign by expert radiologists through manual assessments [11]. However, this approach can affect variations in expertise among radiologists, repeating issues, and subjectivity. Additionally, the accuracy of artificial ultrasound detection is affected by the high noise and low resolution of the ultrasound images [12]. Thereafter, automated tumor classification became very popular to overcome these difficulties.

Image classification, a key concept in pattern recognition and computer vision, involves assigning labels to images. Traditionally, this concept involves extracting features from the image, typically low-level or mid-level, and then utilizing a trainable classifier for labeling. However, in the past few years, deep learning networks with convolutional layers have emerged as superior, offering high-level feature representations compared to manually crafted features [13]. Originally developed to classify images, deep learning networks with convolutional layers are fundamental for image processing [14]. They utilize successive convolution and pooling layers. Despite their classification accuracy, Convolutional Neural Networks (CNNs) may experience performance decline due to reduced data dimensionality for spatial invariance, leading to the loss of crucial information, such as rotation and scale attributes. This loss impacts the effectiveness of segmentation, object detection, and localization accuracy [15]. Since breast cancer is a heterogeneous disease clinically [16], breast cancer classification systems have been developed to standardize the language and organize this heterogeneity [17].

In this research, we present a hybrid deep learning model based on an enhanced U-Net and Capsule Network (called UCapsNet) to enhance the detection and classification of breast cancer in ultrasound imaging. The hybridization of U-Net and capsule networks into UCapsNet addresses critical gaps in current breast cancer detection methods. Traditional U-Net models often lose important spatial details due to pooling layers, which can reduce segmentation precision. However, capsule networks excel in preserving spatial hierarchies and enhancing classification accuracy through dynamic routing. By combining U-Net’s segmentation strength with the capsule networks’ classification capabilities, the proposed UCapsNet model improves both tumor boundary delineation and differentiation between benign and malignant tumors. Our UCapsNet model addresses critical gaps in current breast cancer detection methods, to improve both segmentation and classification accuracy through the integration of U-Net and capsule networks. The key contributions of the proposed UCapsNet model presented in this paper can be summarized as follows:

- We introduce UCapsNet, a hybrid deep learning model that utilizes an enhanced U-Net for segmentation and capsule networks for robust classification.
- Our enhanced U-Net model incorporates increasing filter counts, adding dropout layers and employing skip connections, which enable more precise segmentation results in poor-quality ultrasound images.
- The use of capsule networks replaces traditional pooling layers, allowing for the preservation of spatial relationships often lost in standard models. Using dynamic routing between capsules, the proposed model shows an enhanced ability to differentiate between benign and malignant tumors.
- By merging the segmentation capabilities of U-Net with the classification power of capsule networks, we overcome the drawbacks of the existing techniques related to the loss of spatial detail and improve diagnostic performance.
- We conduct extensive evaluations of UCapsNet against well-known pre-trained models, including VGG-19, DenseNet, MobileNet, ResNet-50, and Xception.

## 2. Related Works

### 2.1. Preprocessing Techniques for Ultrasound Image Enhancement

Preprocessing techniques are essential for enhancing ultrasound image quality, often degraded by noise, low contrast, and artifacts. Techniques like histogram equalization, used by Asadi et al. [18], improve image contrast to support accurate segmentation. Similarly, Zeebaree et al. [19] employed median and Wiener filters to reduce speckle noise, improving texture and reducing overlaps between benign and malignant cases. Benaouali et al. [20] used anisotropic filtering alongside the Level Set Method to refine region of interest (ROI) boundaries, followed by texture-based feature extraction to aid in accurate tumor delineation. While these traditional preprocessing methods improve image clarity and reduce noise, they are limited in handling complex artifacts and variability in ultrasound images, particularly for more intricate tumor structures.

In contrast, UCapsNet leverages preprocessing within an enhanced U-Net architecture, which includes skip connections and increased filter counts. This integrated approach retains essential spatial details that would otherwise be lost, particularly in low-quality ultrasound images. By combining preprocessing with a powerful segmentation model, UCapsNet achieves a level of clarity and robustness in segmentation that traditional filters alone cannot provide.

### 2.2. Segmentation Techniques for Breast Cancer Detection

Accurate segmentation of tumor boundaries in ultrasound images is a critical step in breast cancer detection. U-Net, a widely used architecture for medical image segmentation, is effective for its encoder–decoder structure but suffers from spatial detail loss due to pooling layers. For example, Jui-Ying Bs et al. [21] used Mask R-CNN to automate segmentation and classification by incorporating region proposal techniques. Although this approach improves segmentation accuracy, it can still lose essential spatial details necessary for precise localization. Hekal et al. [22] introduced the Dual-Decoder Attention ResUNet (DDA-AttResUNet), which combines dual decoder attention layers with U-Net, achieving a Dice score of 92.92%. Pramanik et al. [23] developed the Dual-Branch U-Net (DBU-Net) tailored for tumor segmentation in breast ultrasound images. Their method leverages dual branches to capture more nuanced features, achieving competitive segmentation performance. Yan et al. [24] introduced AEU-Net, which uses a Hybrid Dilated Convolution (HDC) model to enhance the accuracy of tumor segmentation in ultrasound images. It reported improved performance on the Dice coefficient. Tong et al. [25] presented an improved U-Net model incorporating Multi-Atlas Label Fusion (MALF) for lesion segmentation in breast ultrasound images. Their method focuses on better handling of spatial features.

Although such models enhance segmentation quality, they often face computational demands, and their accuracy is limited by the structural constraints of traditional CNN architectures. UCapsNet’s enhanced U-Net addresses these challenges by incorporating skip connections and a higher filter count, which helps retain spatial details across network layers, leading to better boundary delineation. This improvement is particularly important in ultrasound imaging, where the noise and low contrast frequently hinder precise segmentation. By preserving fine spatial details, UCapsNet’s U-Net component produces clearer tumor masks that serve as reliable inputs for the classification stage, ultimately improving the model’s diagnostic performance.

### 2.3. Classification Techniques for Tumor Identification

Classification models based on CNN architectures, such as ResNet, DenseNet, MobileNet, and VGG-16, have been widely adopted for distinguishing benign from malignant tumors due to their strong performance in image recognition tasks. For instance, Hijab et al. [26] applied transfer learning with VGG-16, achieving an accuracy of 97%. Jabeen et al. [27] used DarkNet-53 with data augmentation and optimization algorithms, achieving an accuracy of 99.1%. Uysal and Köse et al. [28] applied deep learning-based models for the classification of breast ultrasound images, utilizing models such as ResNet-50 and VGG-16, with a focus on data augmentation and handling class imbalances. This work could be included to emphasize the challenges in balancing accuracy with dataset limitations. Balasubramaniam et al. [29] proposed a modified LeNet CNN for breast cancer diagnosis in ultrasound images. Their model incorporates enhancements like batch normalization and modified ReLU to prevent overfitting, making it a relevant point of comparison in the context of model performance stabilization. Wei et al. [12] used texture and morphological feature extraction combined with classifiers for differentiating benign and malignant tumors in ultrasound images. Texture analysis remains a popular approach, though it often requires manual feature extraction.

Despite these successes, CNNs are limited in retaining spatial hierarchies due to pooling layers, which can result in the loss of subtle morphological details essential for distinguishing complex tumor structures. While transfer learning and data augmentation partially address these issues, CNNs still struggle to retain the spatial context necessary for accurate tumor classification in noisy ultrasound environments. UCapsNet overcomes this limitation by employing a capsule network for classification, preserving spatial hierarchies through dynamic routing. This action enables UCapsNet to maintain spatial relationships within the image, allowing for more accurate differentiation between benign and malignant tumors. Unlike traditional CNNs, which often require extensive data augmentation to generalize well, UCapsNet’s capsule network achieves reliable classification with minimal preprocessing, enhancing the model’s robustness in clinical applications.

### 2.4. Hybrid Approaches for Breast Cancer Detection and Classification

Hybrid models, which integrate both segmentation and classification stages, leverage the strengths of each approach to improve overall accuracy in breast cancer detection. Xiaozhen Xie et al. [30] proposed a model combining ResNet with Mask R-CNN, achieving a high classification precision of 98.72%. Similarly, Junaid Umer et al. [31] developed a multiscale cascaded convolutional network with residual attention-based decoders, achieving a Dice score of 90.55% on segmentation tasks. Lanjewar et al. [32] combined MobileNetV2, ResNet50, and VGG16 with LSTM for feature extraction, addressing class imbalance with SMOTETomek and enhancing interpretability with Grad-CAM. This work is particularly relevant for hybrid architectures that integrate transfer learning models for improved classification. While these methods improve diagnostic accuracy, they often rely on pre-trained CNNs or require separate feature extraction steps, which add to computational complexity. Additionally, supervised texture classification methods, as demonstrated by Liu et al. [33], incorporate texture analysis to improve accuracy, but they are heavily dependent on manual feature extraction and preprocessing.

In contrast, UCapsNet represents a hybrid model that integrates segmentation and classification seamlessly by combining an enhanced U-Net with a capsule network. Unlike previous hybrid models that rely on pre-trained CNNs or require additional feature extraction steps, UCapsNet’s two-stage approach directly feeds precise segmentation results into the capsule-based classifier. This streamlined integration reduces computational overhead and improves diagnostic performance, making UCapsNet a practical and efficient choice for clinical breast cancer detection and classification in ultrasound imaging. Table 1 provides a summary highlighting the findings of pivotal works.

## 3. Data Collection

**Figure 1:**
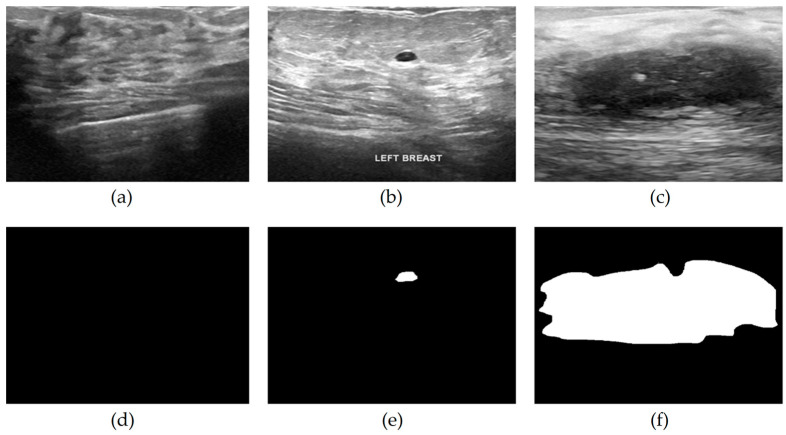
Dataset image samples: (**a**) normal ultrasound image, (**b**) benign ultrasound image, (**c**) malignant ultrasound image, (**d**) masked image of (**a**), (**e**) masked image of (**b**), (**f**) masked image of (**c**).

The BUSI Dataset [36], used for training and evaluating the proposed model, includes 780 grayscale ultrasound images in DICOM format which are later converted to PNG format. Originally, 1100 images were gathered over a year at Baheya Hospital in Egypt. Following preprocessing to eliminate irrelevant information that could impact classification accuracy, the dataset was refined to its current 780 images, categorized as normal (133 images), benign (437 images), and malignant (210 images). These images have a resolution of 500 × 500 pixels, though the original scan resolution was 1280 × 1024, achieved using the LOGIQ E9 and LOGIQ E9 Agile ultrasound systems. These advanced imaging systems, often used in radiology, cardiac, and vascular applications, employed ML6-15-D Matrix linear probes operating at 1–5 MHz, providing high-resolution images well suited for detailed lesion analysis. In addition to the ultrasound images, the BUSI dataset includes segmentation masks that delineate lesion areas, aiding in accurate lesion localization during model training. The dataset reflects the regional demographic, with samples from 600 female patients aged 25 to 75. Although it is valuable for developing breast cancer detection and classification models, the dataset does present certain limitations, including limited demographic diversity and a relatively small sample size compared to other medical imaging datasets. These factors may affect the generalizability of models trained solely on this dataset, particularly when applied to broader and more varied populations. Sample images from each class and their corresponding masks are presented in Figure 1.

## 4. Materials and Methods

**Figure 2:**
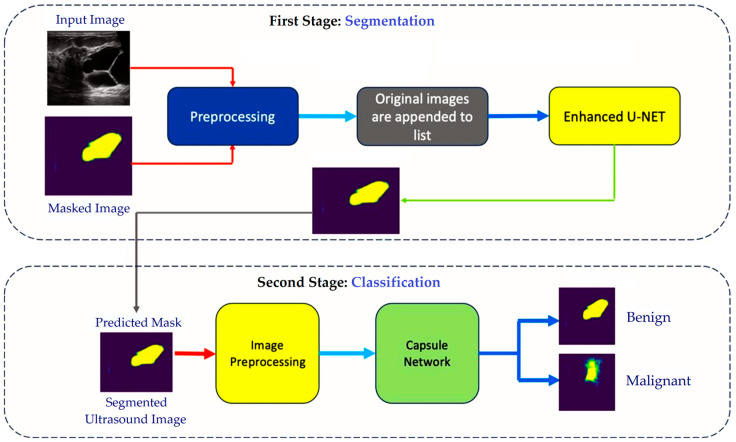
Workflow diagram of the segmentation and classification parts within the proposed two-stage UCapsNet model.

The proposed UCapsNet model was implemented using Python 3.10. The overall workflow of the proposed two-stage UCapsNet model involves a structured series of steps to ensure the accurate segmentation and classification of breast cancer from ultrasound images. As shown in Figure 2, these steps illustrate how the segmentation and classification stages are carefully structured to tackle the unique challenges of breast ultrasound imaging. The workflow can be broken down as follows:

- Stage 1 (Segmentation): In the first stage, the model focused on identifying and isolating the tumor region within the ultrasound image using an enhanced U-Net, specifically designed to address the challenges of noise, variability, and lower quality often found in ultrasound data. The key improvements to the standard U-Net include increased filters in the convolutional layers, enabling the model to capture finer details and subtle variations crucial for accurately defining the tumor edges. Additionally, skip connections were incorporated between the encoder and decoder layers, helping to preserve essential high-resolution features that might otherwise be lost during segmentation. Together, these enhancements allowed the model to produce a clear, precise outline of the tumor, which then served as input for the next classification stage.
- Stage 2 (Classification): After segmentation, the classification stage used a capsule network to distinguish between benign and malignant tumors. The capsule network is particularly suited for this task as it maintained the spatial relationships within the image, which is essential for accurately characterizing breast tumors. Unlike traditional convolutional networks that may lose spatial details through pooling, the capsule network preserved these details, enabling it to capture the subtle textural and structural differences between tumor types. Additionally, capsule networks were designed to recognize objects regardless of orientation or slight distortions, which means they can reliably classify tumors based on their intrinsic patterns rather than being thrown off by minor image variations. In this stage, the capsule network analyzed the segmented tumor region. It made the final classification, identifying whether the tumor is benign or malignant based on the spatial features it learned.

The proposed combined two-stage approach of using an enhanced U-Net model for segmentation and a capsule network for classification enabled each stage to enhance the other, creating a cohesive and reliable model. By isolating the tumor in Stage 1 and classifying it with spatial accuracy in Stage 2, UCapsNet effectively addressed the diagnostic challenges specific to breast ultrasound images. This method capitalized on the strengths of both networks to achieve a robust and accurate tool for breast cancer detection, making the proposed UCapsNet model a reliable and effective tool for detecting breast cancer in ultrasound images.

### 4.1. Segmentation

#### 4.1.1. Data Preprocessing

This research introduces an improved model for segmenting tumor regions in BUSIs. To start, all images were resized to 256 × 256 pixels. A dictionary was then created with two lists: one for the resized original images and another for their corresponding ground truth masks. The proposed enhanced U-Net generated predicted masks, which were compared to the ground truth masks for evaluation.

#### 4.1.2. System Architecture of Segmentation

**Figure 3:**
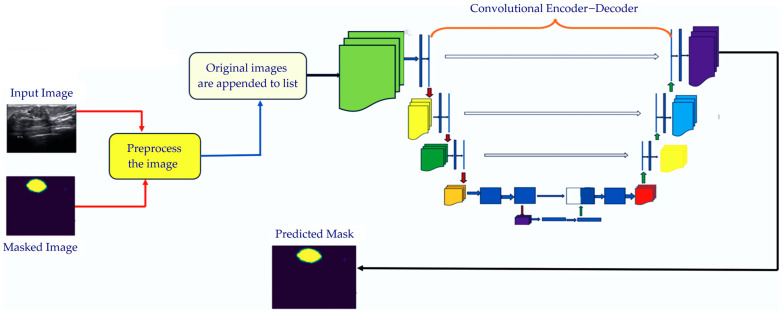
Illustration of the enhanced U-Net architecture.

The original image was then given to the enhanced U-Net architecture and a binary mask was generated by the model. The architecture of the enhanced U-Net, as shown in Figure 3, has four encoder blocks, a bottleneck layer, and four decoder blocks. The encoder block contained two 3 × 3 convolutional layers one after the other with ReLU as its activation function and He-normal initialization. These convolutional layers were responsible for the extraction of the features. After the first convolution, batch normalization was added. After each encoding block, we added a pool size max-pooling layer of 2 × 2. In the model’s encoder part, it performed down-sampling, which resulted in lower-dimensional feature maps. At the tail of the encoder part was a bottleneck layer consisting of two 3 × 3 convolutional layers one after the other with ReLU as its activation function, and then, we added batch normalization. Initially, the encoder part started with 64 filters, and they were doubled at every convolution layer. In the decoder part, we added the convolutional Transpose layers to up-sample the fewer dimensional feature maps in our proposed model. Between the decoder and encoder parts of the network, we implemented skip connections to ease the flow of information and bypass unnecessary layers when appropriate. This framework enabled the network to extract small and highly influential features that may not be feasible to obtain through traditional methods. However, by skipping the connections, the network can combine features of lower levels from the encoder layers with features of higher levels from the decoder layers, resulting in an accurate segmentation of tumor regions.

By offering a more robust and reliable method for breast cancer tumor segmentation, this approach enhanced the overall performance of the U-Net model. At each decoder block, we used two 3 × 3 convolutional layers one after the other with ReLU as the activation function, using He-normal initialization. After this decoder block, dropout layers wre added. Finally, we utilized a 1 × 1 convolutional to perform as the output layer, which used the activation function sigmoid and gave a binary mask as the output. The number of filters in the enhanced U-Net is the key difference between it and the U-Net model, where it started with 64 filters, and the filters were doubled gradually as we went deeper into the network. Another difference is that we also added extra dropout layers to prevent overfitting. The enhanced U-Net includes several enhancements that made it more powerful and capable of capturing complex patterns in the data. The binary masks produced by the Enhanced U-Net model were given as the input to the capsule network for classification.

### 4.2. Classification

#### 4.2.1. System Architecture of Classification

**Figure 4:**
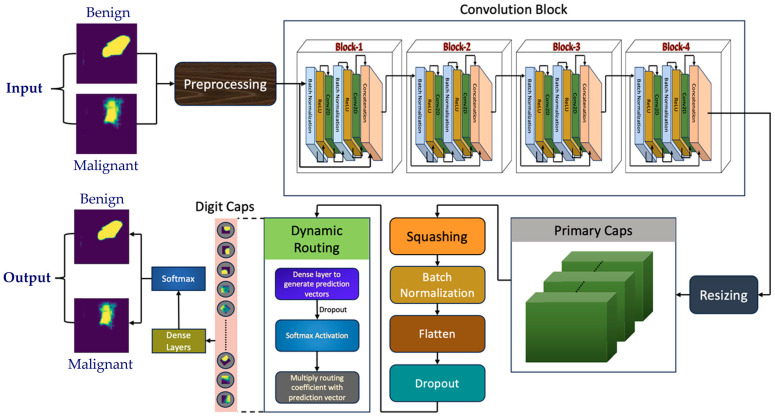
Illustration of the proposed capsule network.

Figure 4 depicts the structure of the suggested capsule network for categorization. The preprocessing began by randomly shuffling the images and the labels in the same order. The proposed capsule network expected the input of dimensions (None, 128, 128, 3), meaning a batch of images of size 128 × 128 pixels was taken as the input with three color channels (RGB). However, the predicted masks by the enhanced U-Net were of dimensions (None, 256, 256, 1). The predicted masks were resized to a smaller size of (128 to 128) to reduce the computational complexity and enhance the model’s training efficiency by working with smaller input images. After resizing, the masks were expanded to include a channel dimension and were repeated three times to form three-channel images. This adjustment aligns with the input shape expected by the capsule network model, which required images in RGB format.

Following these steps, the input dimensions were (None, 128, 128, 3), meeting the requirements of the capsule network. In the next phase, zero padding was applied to the input, expanding its dimensions to (None, 134, 134, 3), where additional rows and columns filled with zeros were added around the input. This padding helped preserve the spatial dimensions of the input when it was passed to the convolution layer. The padded input was then fed into a 2D convolutional layer with 64 filters. After the convolution operation, the dimensions were reduced to (None, 64, 64, 64), with 9408 parameters to be trained. Batch normalization was executed following the Convolution Layer to normalize its output. By performing this step, the training process was accelerated. Next, an activation function, ReLU, was applied to introduce decorrelation to the model, capturing more complex and nonlinear relations within the input data. Another zero-padding layer was applied to the output of the activation function, resulting in a shape of (None, 66, 66, 64). For further processing of the data, the dimensions were then reshaped to (None, 32, 32, 64); by doing so, the most important features were extracted and were further processed by the network.

#### 4.2.2. Convolution Block

**Figure 5:**
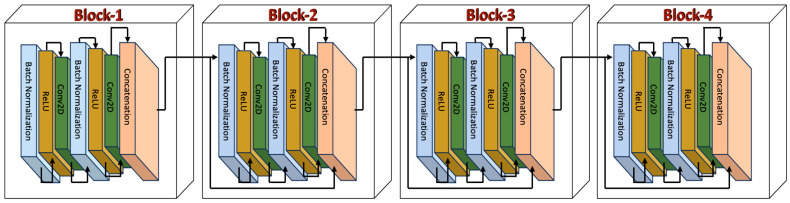
Illustration of the convolution block in the proposed model.

Most CNNs utilize alternating convolution and pooling layers for feature extraction, followed by fully connected layers for classification. However, pooling layers can result in the loss of important information about objects in images. To overcome this challenge, we integrated the DenseNet model, which connected multiple convolutional blocks to enhance feature extraction (as shown in Figure 5). First, the output from previous layers, which have the dimensions of (None, 32, 32, 64), was fed into a 2D convolutional layer. This layer helped the model to learn more detailed, layered representations of the input. Then, the output was reshaped into (None, 32, 32, 128) and introduced 8192 learnable parameters, enabling the model to capture even more nuanced features of the data. Next, batch normalization and an activation layer, respectively, were applied to refine the features. The batch normalization layer had 512 parameters per feature map. The output generated after these layers has the dimension (None, 32, 32, 128). Subsequently, another 2D Convolutional layer was utilized, which generated 32 feature maps of size 32 × 32, utilizing 36,864 parameters. So, the output generated had the dimension (None, 32, 32, 32). The feature maps from different parts of the network were then concatenated along the channel axis for integrating diverse features and learning complex representations in the data. The concatenated data had the dimension (None, 32, 32, 192), which means it generated 192 feature maps of size 32 × 32. The same process mentioned was performed in the consequent three blocks as well.

#### 4.2.3. Capsule Block

The extracted features from the convolutional block were then divided into partitions, referred to as primary capsules, which encoded information about specific entities identified by the convolutional layers. Moving forward, the subsequent layer comprised secondary capsules, where each partition in the primary capsules attempted to predict the output in the following layer. This predictive process, known as dynamic routing, facilitated the flow of information between the capsules. During dynamic routing, the predicted information from individual capsules was compared with the original information. If there was disagreement in the predictions among the capsules, the weights were decreased; conversely, if agreement was reached, the weights were increased to a sustainable level. This iterative process is termed routing by agreement.

The primary capsule layer within the capsule network framework serves as a pivotal component for feature extraction and representation from the input data. This layer received the feature maps from the preceding intermediate model created by using DenseNet121 and reshaped features into vectors known as capsules by applying a series of operations. As a result of these operations, the feature vectors were resized to 3-dimensional tensors. These capsules were designed to encode diverse attributes and characteristics of the input data, enabling robust and hierarchical feature learning within the network and preserving semantic information and spatial relationships. Subsequently, the squash function was applied to the transformed tensor. This squash function was the activation function used to bring nonlinearity for standardizing the vectors and ensured that their lengths fell between 0 and 1 while conserving vector magnitude. For all the capsules in the primary capsule layer, this squash function was applied, which results in the output vectors with hierarchical entity features. This standardization facilitated efficient feature representation by constraining vector magnitudes, thereby preventing dominance by larger activations and fostering a balanced distribution of information across capsules. For an input vector *s_j_*, the squash function is mathematically expressed as follows:

(1) $$squash\left(s_{j}\right)=\frac{{∥s_{j}∥}^{2}}{1+{∥s_{j}∥}^{2}}\;\cdot \;\frac{s_{j}}{∥s_{j}∥+\epsilon }\;$$

where

- $∥s_{j}∥$: Euclidean Magnitude of the input vector.
- $\frac{s_{j}}{∥s_{j}∥}$: Normalization of v to calculate the unit vector $\hat{s_{j}}$.
- $\epsilon =1\times 10^{-7}$.

In the dynamic routing algorithm, the output vectors from the primary capsule layer served as inputs for an iterative routing process. First, batch normalization was performed to standardize the input vectors for improving convergence and stabilizing training. The normalized capsules were then flattened to continue further processing. Dropout regularizations were added frequently to avoid overfitting and improve generalization. Next, for each capsule, predictions were calculated by passing the flattened capsules through fully connected dense layers with the ReLU activation function. These predictions were used for calculating the routing coefficients. To govern the contribution of every capsule towards the eventual prediction, these coefficients were used. The routing coefficients were calculated using the SoftMax function; this step ensured that the sum of all routing coefficients of the capsules is 1, and they represented the distribution of probability among the capsules. For refining the predictions, at each iteration of the routing process, SoftMax was applied. Once the routing coefficients were computed, they were multiplied by the prediction vectors and weights, resulting in the aggregation of information from all capsules based on their significance. Then, for detecting more complex features, parametric ReLU was applied, which introduced nonlinearity. The above process was iteratively repeated multiple times until the capsules reached a conclusion based on their predictions. For further refinement, the output from the dynamic routing was given to some additional dense layers accompanied by a SoftMax layer for generating the prediction.

The SoftMax activation function played a major role in the computation of routing coefficients based on the input capsules and agreement between the predicted vectors. This function ensured that always valid probabilities were represented by the routing coefficients, which assisted the capsules in concluding fruitfully. The SoftMax activation function took a vector of outputs from a network and gave a vector of scores of probabilities. The SoftMax activation function is mathematically expressed as follows:

(2) $${softmax\left(\vec{x}\right)}_{i}=\frac{e^{x_{i}}}{\sum_{j=1}^{n}e^{x_{j}}}$$

where

- $\vec{x}$: The vector is given as input.
- $e^{x_{i}}$: Input Vector given to Standard Exponential Function.
- n: In a multi-class classifier, the number of classes.
- $e^{x_{j}}$: Output Vector given to Standard Exponential Function.

The loss function was utilized by the network while training for learning more distinct features and for making predictions accurately. It works on the principle of margin-based classification. The margin-loss function’s role was to ensure that the appropriate capsule associated with a specific object in the image was adequately activated while also preventing excessive interference from other capsules. This step was achieved by comparing capsule activations against predetermined margins, akin to thresholds. If the activation of the suitable capsule fell below a certain threshold or if other capsule activations exceeded it, the margin loss function prompted adjustments to the network’s parameters to enhance classification accuracy. The loss function is articulated mathematically as follows:

(3) $$L\left(y,\hat{y}\right)=\sum_{k}y_{k}{max(0,m^{+}-\hat{y_{k}})}^{2}+\lambda \sum_{k}\left(1-y_{k}\right){max(0,\hat{y_{k}}-m^{-})}^{2}$$

where

- $y_{k}$: Represents the true label (ground truth) for class *k*.
- $\hat{y_{k}}$: Represents the predicted probability for class *k* generated by the model.
- $m^{+}$ and $m^{-}$: Margin parameters for positive and negative classes, respectively.
- λ: Regularization parameter.

## 5. Results and Comparisons

The environment for the execution of these experiments used 13 GB of Random-Access Memory along with 2 Intel Xenon CPUs, Python 3.10, and an NVIDIA Tesla K80 graphical processing unit with 12 GB of Random-Access Memory. The model undergoes training for a total of 50 epochs, with a batch size of 25 and a learning rate of 0.00001, utilizing the Adam optimizer for model enhancement.

### 5.1. Segmentation Results

In this section, we review the results of our enhanced U-Net model and compare it with other models.

#### 5.1.1. Results of Proposed Enhanced U-Net

**Figure 6:**
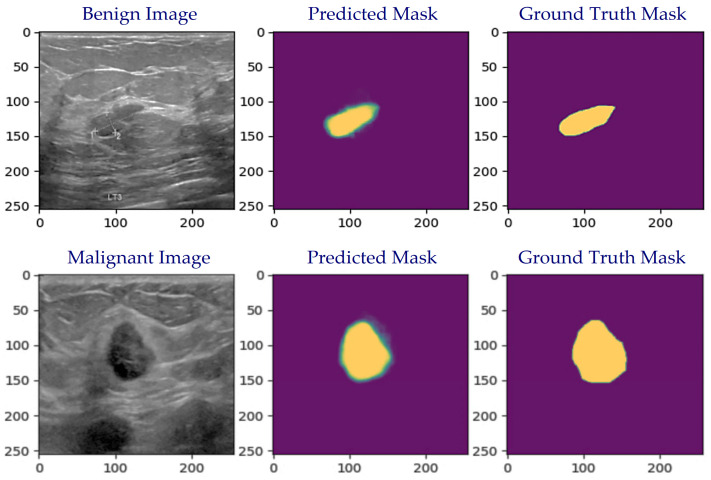
Examples of the original image vs. predicted mask and ground truth mask.

Figure 6 shows the output of our model, which contains the actual ultrasound image from a dataset, the predicted mask generated by our model, and the ground truth mask from the dataset.

#### 5.1.2. Comparison with Other Techniques

**Figure 7:**
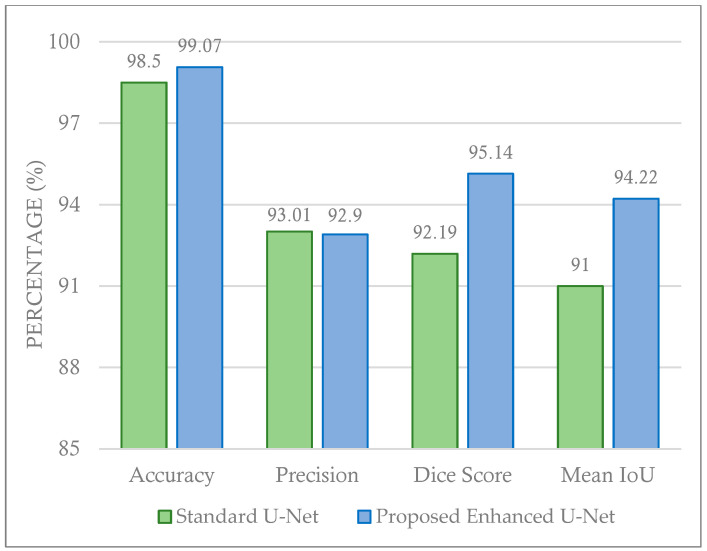
Comparing the proposed enhanced U-Net model with standard U-Net.

**Figure 8:**
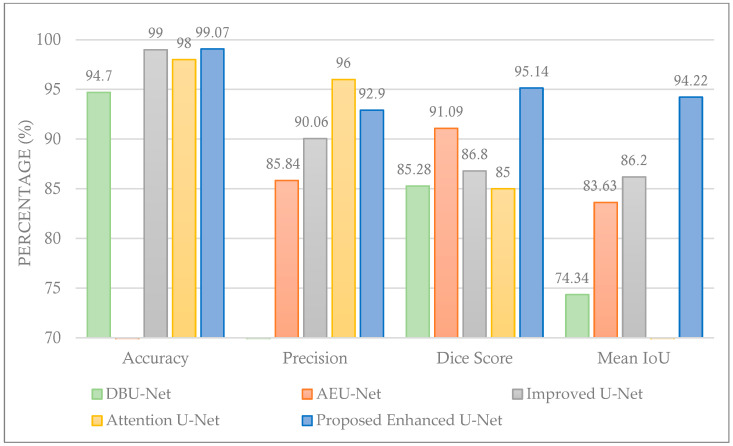
Comparing the proposed enhanced U-Net model with other models.

To evaluate the proposed model’s effectiveness, the results in Table 2 and Figure 7 and Figure 8 are used to compare the proposed enhanced U-Net model with the other models. The evaluation of the proposed enhanced U-Net model’s performance is based on several key metrics, including accuracy, precision, dice coefficient, and mean IoU. In Table 2, the best result for each metric across all techniques is highlighted in bold. These metrics were chosen to provide the assessment of both the segmentation quality and overall effectiveness of the model. As can be found in Table 2, the proposed enhanced U-Net model outperforms the standard U-Net (on average) as well as other U-Net variations, demonstrating its robustness and accuracy.

In terms of accuracy, the proposed enhanced U-Net achieved 99.07%, surpassing the standard U-Net’s 98.50%. This small but significant improvement highlights the model’s increased ability to correctly segment tumor regions. The precision obtained was 92.90% (a bit worse than the standard U-Net with 93.01%), reflecting the proposed model’s ability to reduce false positives compared to other U-Net models. The dice coefficient, which measures the overlap between the predicted and actual tumor regions, rose to an impressive 95.14%, a clear improvement over the standard U-Net’s 92.19%. This improvement suggests that the proposed enhanced U-Net more accurately delineates tumor boundaries, a critical aspect in medical imaging tasks. Finally, the mean IoU was 94.22%, again significantly higher than the standard U-Net’s 91.00%. These results demonstrate that the proposed enhanced U-Net model outperforms existing models across multiple dimensions. The higher accuracy, precision, Dice score, and mean IoU indicate that the improvements made to the U-Net architecture—such as increased filter counts and added skip connections—play a crucial role in refining both segmentation quality and diagnostic reliability. These findings suggest that the proposed model is not only more effective in detecting tumors but also more reliable for practical applications in clinical settings, potentially improving the early detection and treatment of breast cancer.

### 5.2. Classification Results

In this section, the performance of the capsule network is evaluated through the classification of the segmented images derived by the enhanced U-Net model.

#### 5.2.1. Results of the Proposed Model

The performance of the proposed Capsule Network is evaluated using 5-fold cross-validation on the segmented images produced by the enhanced U-Net, classifying these images as either malignant or benign. In this process, the dataset is divided into five parts, and, in each fold, one part is set aside for testing while the remaining parts are used for training. This approach ensures a thorough evaluation across different portions of the data. The hyperparameter settings for the model are detailed in Table 3.

**Figure 9:**
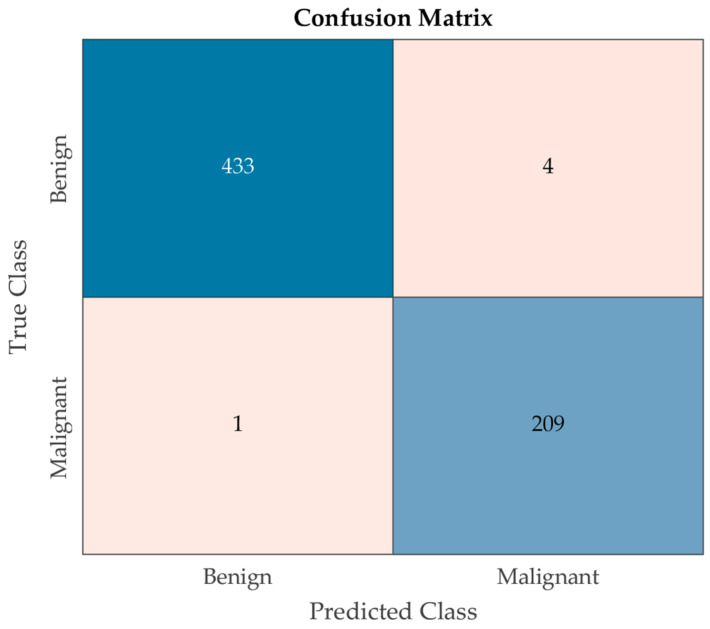
Confusion matrix of the proposed model on the test dataset.

Figure 9 presents the confusion matrix, which further confirms the model’s high classification accuracy. The matrix reveals a balanced distribution of correctly predicted benign and malignant cases, with very few misclassifications, underscoring the model’s robustness in real-world clinical settings. Another point is that the False-Positive Rate (FPR) for benign tumors is significantly lower than that for malignant tumors, which is particularly valuable for early detection and treatment planning.

The performance of the proposed UCapsNet model on the test dataset is summarized in Table 4. The obtained results showcase its high precision, recall, F1-Score, and accuracy for both benign and malignant tumor classifications. The results highlight the model’s excellent ability to generalize unseen test data samples with high accuracy and reliability. For benign tumors, the model achieved an accuracy of 99.22%, with a precision of 99.76%, a recall of 99.08%, and an F1-Score of 99.42%. These results demonstrate the model’s exceptional capability to correctly identify benign tumors while keeping false positives low. Similarly, for malignant tumors, the model delivered strong results, achieving 99.22% accuracy, 98.12% precision, 99.52% recall, and a 98.81% F1-Score. These findings confirm that the UCapsNet model is a highly effective tool for accurately distinguishing between benign and malignant tumors, making it a valuable asset in clinical breast cancer diagnosis.

#### 5.2.2. Comparison with Pre-Trained CNNs

A comparison of the results of the proposed model with five well-known pre-trained CNN models (VGG-19, ResNet-50, MobileNetV2, DenseNet121, and Xception) can be seen in Table 5, wherein the best result in each column highlighted in bold. The results demonstrate that our model significantly outperforms all other CNN models during training and testing. It achieved an impressive 99.58% accuracy in the training phase and 99.22% in the test phase, with minimal training and test losses of 0.007% and 0.02%, respectively. This result shows the proposed model’s efficiency and reliability in real-world applications. This enhanced accuracy highlights the model’s effectiveness in handling complex breast cancer detection tasks, which offers a significant improvement over traditional CNN models.

#### 5.2.3. Comparison with Existing Techniques

**Figure 10:**
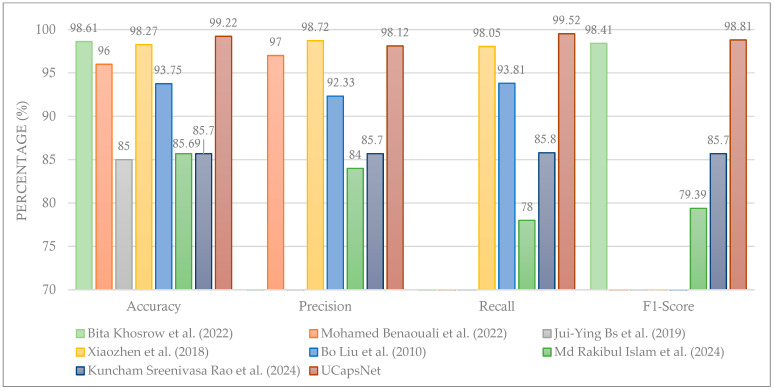
Comparing the proposed UCapsNet model with existing techniques [18,20,21,30,33,34,35].

A comparison of the proposed UCapsNet model with existing techniques from the literature is provided in Table 6, wherein the best result for each performance measure is shown in bold. Furthermore, a visual comparison of the different techniques is provided in Figure 10. The results demonstrate that the proposed UCapsNet model stands out by achieving the highest accuracy (99.22%) among all compared models. In contrast, other models like ResNet-50 used by Bita Khosrow et al. [18] achieved a slightly lower accuracy at 98.61%, while traditional machine learning techniques, such as SVMs and decision trees used by Mohamed Benaouali et al. [20], reached 96%. The results indicate UCapsNet’s superior ability to correctly classify both benign and malignant breast cancer tumors.

### 5.3. Discussion

The obtained results from our experiments provide a comprehensive evaluation of the proposed UCapsNet model for breast cancer segmentation and classification using ultrasound images. For the segmentation task, our enhanced U-Net model showed impressive results, with an accuracy of 99.07%, a Dice score of 95.14%, and a mean IoU of 94.22%. These improvements came from adding extra filters and optimizing the skip connections in the U-Net architecture. On the classification side, the proposed UCapsNet model delivered exceptional performance, achieving a test accuracy of 99.22% for tumor classification. The obtained classification results demonstrate the capability of the proposed model to detect benign and malignant cases with high reliability. One of the key strengths of the model is its ability to reduce false negatives for malignant tumors, which means it is less likely to miss identifying a cancer case. This finding makes the model particularly valuable in real-life medical settings, where catching these cases early is critical.

The superior performance of the proposed UCapsNet model in terms of precision, recall, accuracy, and F1-Score, underscores its potential as a State-of-the-Art tool for breast cancer diagnosis. The proposed model demonstrates remarkable improvements in both segmentation and classification tasks. These results suggest that our model could play a vital role in improving early breast cancer detection and treatment planning, offering a reliable, high-performing solution that outpaces techniques in the literature.

## 6. Potential Implications, Limitations, and Future Research

UCapsNet’s strong performance in segmentation and classification has exciting potential for clinical practice, especially in improving early breast cancer detection. By precisely identifying and outlining tumor regions, this model could enable earlier interventions, leading to better outcomes and allowing for less invasive treatment options. It could also help reduce differences in diagnostic results among radiologists, which is particularly valuable in settings with limited access to specialized expertise. By bringing consistency to ultrasound interpretations, UCapsNet could support reliable diagnostics across a wide range of healthcare facilities. Additionally, by automating the segmentation and classification steps, it can save radiologists time on manual processing, allowing them to dedicate more attention to in-depth case reviews. Altogether, UCapsNet shows promise as a practical tool to enhance the accuracy and accessibility of breast cancer diagnostics in clinical settings.

Despite the significant advancements of the proposed UCapsNet model for breast cancer segmentation and classification from ultrasound images, several limitations remain. Firstly, limitations in the dataset affect the model’s generalizability. This study used the BUSI dataset, which, while well regarded, may not fully capture the diversity of imaging conditions and tumor variations found in real-world applications. Expanding UCapsNet’s evaluation to include diverse datasets—encompassing a range of demographics, imaging devices, and clinical settings—would enhance its robustness. Furthermore, the proposed model was tested solely on this dataset, without evaluation on independent datasets, which limits insights into its generalizability. Secondly, UCapsNet’s computational complexity, especially within its capsule network component, presents challenges for real-time clinical deployment. Capsule networks require significant computational resources due to their complex dynamic routing mechanisms, which may impact processing speeds. Future studies could focus on developing efficient capsule network variants or exploring optimization techniques, such as model pruning or quantization to enhance UCapsNet’s suitability for resource-constrained environments. Another limitation involves the model’s sensitivity to noise and artifacts often present in ultrasound images. While UCapsNet shows improved accuracy, it may still be affected by lower-quality images with high levels of noise or occlusions. Incorporating advanced noise reduction methods or training the model with synthetic noise patterns could improve its robustness in these conditions.

For future work, metaheuristic-driven optimization techniques (e.g., genetic algorithms, particle swarm optimization, and gray wolf optimizers) could be used for hyperparameter tuning to further enhance the performance of the UCapsNet model. Furthermore, ensemble deep learning techniques can be used at the final layer of the classification stage to reduce the sensitivity of the model to noise and enhance the classification accuracy. As another future work, the image database can be expanded to provide a more diverse set of ultrasound images, which ultimately enhances the generalizability and robustness of the detection model. Additionally, employing data augmentation or domain adaptation methods would help prepare the model for a broader range of imaging conditions and anomalies, which can make the model more adaptable to real-world clinical environments. Finally, incorporating interpretability techniques, such as Grad-CAM or saliency mapping, could offer clinicians insights into the model’s decision-making process, increasing the likelihood of its acceptance in clinical practice.

## 7. Conclusions

In this paper, we introduced UCapsNet as a hybrid two-stage deep learning framework that integrates an enhanced U-Net with a capsule network for the automation of breast lesion segmentation and classification from ultrasound images. The proposed model combines the robust segmentation capabilities of U-Net with the spatial hierarchy preservation and dynamic routing features of capsule networks. By enhancing the segmentation process and improving the classification accuracy, the UCapsNet model addresses the limitations of traditional methods and offers a significant advancement in breast cancer diagnostics. This approach addresses common issues with traditional methods, like the loss of spatial information and reduced accuracy in complex cases, making UCapsNet a significant step forward in breast cancer diagnostics. The simulation results demonstrate that UCapsNet outperforms existing techniques by achieving a superior performance in both segmentation and classification tasks. The model’s precision, recall, and overall accuracy surpass those of conventional pre-trained models and existing techniques. This improved performance highlights UCapsNet’s potential to enhance the reliability and consistency of breast cancer detection from ultrasound images. These findings highlight UCapsNet’s potential to be a valuable tool in clinical settings, supporting early detection and aiding treatment planning.

## Figures and Tables

**Table 1:** Summary of the existing techniques.

Authors	Method	Techniques	Results
Bita Khosrow Asadi et al. [18]	U-Net + ResNet-50	Histogram equalization, CNN with ReLU and SoftMax	Accuracy: 98.61% F1-Score: 98.41%
Zeebaree et al. [19]	Median and Wiener Filters	Removes noise, minimizes overlap in benign/malignant cases	Accuracy: 98.8%
Mohamed Benaouali et al. [20]	Multi-stage Model	Anisotropic filtering, level set method for segmentation	Accuracy: 96% Sensitivity: 97% Specificity: 94%
A. A. Hekal et al. [22]	DDA-AttResUNet	Dual decoder attention for segmentation, intersection over union (IoU), precision	Dice: 92.92% IoU: 87.39% Precision: 93.90%
Ahmed Hijab et al. [26]	VGG-16 CNN	Transfer learning for classification	Accuracy: 97% AUC: 98%
Kiran Jabeen et al. [27]	DarkNet-53 with Transfer Learning	Gaussian walk, differential evolution optimization	Accuracy: 99.1%
Xiaozhen Xie et al. [30]	ResNet with Mask R-CNN	Transfer learning, segmentation, and classification	Precision: 98.72% Recall: 98.05%
Madhusudan G. Lanjewar et al. [32]	MobileNetV2, ResNet50, and VGG16 with LSTM for Feature Balancing	SMOTETomek for data balance, Grad-CAM, and LIME for interpretability	F1-Score: 99.0% AUC: 1.0
Bo Liu et al. [33]	Supervised Texture Classification	Two-stage ROI generation and classification	Accuracy: 93.75%
Md Rakibul Islam et al. [34]	EDCNN Model	Integrated MobileNet and Xception model	Accuracy: 85.69%
Kuncham Sreenivasa Rao et al. [35]	Inception V3 with Stacking Model	Transfer learning with VGG-16	AUC: 94.7% F1-Score: 85.7%

**Table 2:** Comparison of proposed enhanced U-Net vs. standard U-Net.

Model	Accuracy	Precision	Dice Score	Mean IoU
Standard U-Net	98.50%	**93.01%**	92.19%	91.00%
DBU-Net [23]	94.70%	-	85.28%	74.34%
AEU-Net [24]	-	85.84%	91.09%	83.63%
Improved U-Net [25]	99.00%	90.60%	86.80%	86.20%
Attention U-Net [37]	98.00%	96.00	85.00	N/A
Proposed Enhanced U-Net	**99.07%**	92.90%	**95.14%**	**94.22%**

**Table 3:** Hyperparameters of the proposed model.

Parameters	Value
Learning Rate	0.005
Optimizers	Adam
Loss Function	Margin Loss
Epochs	50
Batch Size	25
Epsilon	1 × 10^−7^
Dropout	0.275
Kernel Initializer	Glorot normal

**Table 4:** Performance of the proposed UCapsNet model on the test dataset.

Class	Accuracy	Precision	Recall	F1-Score
Benign	99.22%	99.76%	99.08%	99.42%
Malignant	99.22%	98.12%	99.52%	98.81%

**Table 5:** Comparison of the proposed model with pre-trained CNN models.

Model	Train		Test	
	Accuracy	Loss	Accuracy	Loss
VGG-19	83.84%	0.35%	82.39%	0.39%
ResNet-50	69.99%	0.61%	66.92%	0.65%
MobileNetV2	83.94%	0.72%	80.76%	0.92%
DenseNet121	84.71%	0.34%	83.84%	0.37%
Xception	93.61%	0.15%	88.46%	0.34%
Proposed UCapsNet Model	**99.58%**	**0.007%**	**99.22%**	**0.02%**

**Table 6:** Comparing the performance of the proposed vs. models from the other literature.

Method	Model	Accuracy	Precision	Recall	F1-Score
Bita Khosrow et al. [18]	ResNet-50	98.61%	-	-	98.41%
Mohamed Benaouali et al. [20]	SVM, KNN, Decision trees	96%	97%	-	-
Jui-Ying Bs et al. [21]	Mask R-CNN	85%	-	-	-
Xiaozhen et al. [30]	ResNet with Transfer Learning	98.27%	**98.72%**	98.05%	-
Bo Liu et al. [33]	Kernel SVM	93.75%	92.33%	93.81%	-
Md Rakibul Islam et al. [34]	EDCNN	85.69%	84%	78%	79.39%
Kuncham Sreenivasa Rao et al. [35]	Inception V3 with Stacking	85.7%	85.7%	85.8%	85.7%
Proposed UCapsNet Model	U-Net with Capsule Network	**99.22%**	98.12%	**99.52%**	**98.81%**

## Data Availability

The data that support the findings of this study are openly available online: https://www.kaggle.com/datasets/aryashah2k/breast-ultrasound-images-dataset (accessed on 28 February 2020).
